# Development and evaluation of pharmacist-provided teach-back medication counselling at hospital discharge

**DOI:** 10.1007/s11096-023-01558-0

**Published:** 2023-04-24

**Authors:** E. O’Mahony, J. Kenny, J. Hayde, K. Dalton

**Affiliations:** 1 Pharmacy Department, Tallaght University Hospital, Dublin, Ireland; 2 Pharmaceutical Care Research Group, School of Pharmacy, University College Cork, Cork, Ireland

**Keywords:** Communication, Hospitals, Patient discharge, Patient medication knowledge, Pharmacists, Pharmaceutical services, Teach-back

## Abstract

**Background:**

Pharmacists can use teach-back to improve patients’ understanding of medication; however, the evidence of its impact on patient outcomes is inconsistent. From the literature, there is no standardised way to provide pharmacist-delivered medication counselling at hospital discharge, with limited reporting on training.

**Aim:**

To develop a standardised medication counselling procedure using teach-back at hospital discharge, and to evaluate feedback from patients and pharmacists on this initiative.

**Method:**

A standardised intervention procedure was developed. Participating pharmacists (*n* = 9) were trained on teach-back via an online education module and watching a demonstration video created by the researchers. Pharmacists provided patients with discharge medication counselling utilising teach-back and a patient-friendly list of medication changes to take home. To obtain feedback, patients were surveyed within seven days of discharge via telephone and pharmacists answered an anonymous survey online.

**Results:**

Thirty-two patients (mean age: 57 years; range: 19–91) were counselled on a mean 2.94 medications/patient with the mean counselling time as 23.6 min/patient. All patients responded to the survey, whereby 93.7% had increased confidence regarding medication knowledge and were satisfied with the counselling and the information provided. All pharmacist survey respondents (*n* = 8) agreed they were given adequate training and that teach-back was feasible to apply in practice.

**Conclusion:**

This is the first study to evaluate patients’ views on pharmacist-provided teach-back medication counselling. With positive patient outcomes, a standardised procedure, and a comprehensive description of the training, this study can inform the development of discharge medication counselling utilising teach-back going forward.

**Supplementary Information:**

The online version contains supplementary material available at 10.1007/s11096-023-01558-0.

## Impact statements

- Teach-back medication counselling at hospital discharge increases patients’ satisfaction and confidence regarding medication knowledge.
- Providing written information about medications alongside verbal teach-back counselling from pharmacists is important for patients, and acts as a useful reference point after hospital discharge.
- Pharmacists found this teach-back counselling technique is feasible to deliver at hospital discharge, but the time requirements may necessitate additional staffing.
- Having a finalised discharge medication plan is key before providing teach-back medication counselling prior to hospital discharge.
- This study’s standardised teach-back procedure and the details regarding training should help guide the development of future medication counselling services.

## Introduction

Hospital discharge is a critical healthcare interface and is recognised as a vulnerable time for patients [1]. Medication changes in hospital are common and patients may feel confused if these changes are not communicated to them effectively [2–4]. Indeed, poor communication between healthcare professionals (HCPs) and patients may cause patient harm, involving non-adherence, medication errors, adverse drug events, and rehospitalisations [3, 5, 6].

Pharmacists play a pivotal role in communicating clearly with patients to ensure that they understand what medication changes have been made in hospital, particularly around the time of discharge. There is wide variability worldwide in how this counselling process is conducted at discharge [4, 7]. Many challenges exist which prevent pharmacists from performing discharge medication counselling, such as pharmacists not being informed of intended patient discharge, lack of resources, time limitations, and unexpected discharge [8].

Teach-back is an educational strategy that has been used effectively by pharmacists to assess a patient’s level of medication understanding after counselling [9]. International healthcare organisations recommend teach-back as a universal teaching tool for all patients, and is particularly effective for those with low health literacy [10–12]. Pharmacists who utilised teach-back for medication counselling have demonstrated improvements in patients’ inhaler technique, medication understanding, adherence, patient satisfaction, confidence, and quality of life [13–20]. However, despite these observed benefits, the reported evidence of positive health outcomes has been inconsistent [13, 18–22].

In studies involving pharmacist-led teach-back medication counselling to date, various definitions of teach-back have been used, standardised approaches have been lacking, and most have been conducted in the United States of America [7, 23]. Only a minority reported teach-back training, but specific details were also lacking here [14, 16, 18]. This inconsistency limits the findings’ transferability and generalisability and makes it difficult to inform how pharmacists can provide teach-back in future studies, and makes feasibility in clinical practice difficult to determine. Moreover, reports of pharmacists utilising teach-back for discharge medication counselling are rare [7]. Evidently, there is a need for a standardised teach-back medication counselling process for pharmacists with transparent reporting of teach-back training.

### Aim

This study aimed to fill some of these important gaps by:

(i) Developing a standardised teach-back medication counselling procedure provided by trained pharmacists for patients at hospital discharge, and
(ii) Evaluating pharmacists’ and patients’ feedback on this counselling, including patients’ satisfaction with the intervention and its impact on medication understanding.

### Ethics approval

Ethics approval was initially checked with the St James’s Hospital/Tallaght University Hospital (TUH) Joint Research Ethics Committee; however, this committee deemed this study a quality improvement (QI) initiative and was registered with the TUH QI Lead in March 2021 (registration number 29).

## Method

### Study context and setting

TUH is a 600-bed acute teaching hospital in Dublin, Ireland that manages approximately 17,000 inpatient admissions annually [24]. Pharmacists provide a clinical pharmacy service at ward level by reviewing patients’ drug charts to ensure safe, efficient, economic, and high-quality medication use. At the time of the research, pharmacists were working as part of a consultant-led team under the Collaborative Pharmaceutical Care at Tallaght Hospital (PACT) model [25]. This includes pharmacists performing medication reconciliation and collaborative prescribing of pre-admission medication to ensure the prompt resolution of discrepancies and patients’ safe transition from admission to discharge. Currently, it is not standard practice for HCPs to routinely undertake medication counselling for all patients due to time constraints and limited resources, nor is there any specific training on this. However, pharmacists endeavour to provide medication counselling to inpatients newly commenced on certain high-risk medications, such as anticoagulants, before discharge. A recent survey found that patients discharged from TUH scored the information they received about medication side effects below the national average [26]. Subsequently, TUH management identified this as a high-priority area to improve communication about medication and patient satisfaction on discharge.

### Study design

Study reporting was guided by the Template for Intervention Description and Replication (TIDieR) checklist (Appendix 1, Supplementary Material) [27]. This was a prospective single-centre study to assess the impact of pharmacist-provided teach-back medication counselling at hospital discharge on patient outcomes. It aimed to explore the practicalities of delivering the intervention and to help inform the extent of resources required to utilise this routinely in a busy hospital setting.

### Intervention development, patient recruitment, and data collection

#### Pharmacist training on intervention

Before data collection commenced, pharmacists were trained on the intervention. Education involved completion of an interactive teach-back learning module online that demonstrated the correct integration of teach-back into clinical practice [28]. This educational toolkit is evidence-based and is endorsed by international healthcare institutions. The module was divided into two parts. The first part consisted of a description of teach-back and a demonstration of its effectiveness as a health literacy intervention to improve patient-HCP communication. The second part consisted of a video illustrating a HCP performing teach-back patient education and interactive self-assessment questions asking “*what would I do next?*”. A correct answer confirmed pharmacist understanding and advancement to the next scenario; an incorrect answer resulted in a possible adverse health outcome and the question was repeated until a correct response was achieved. These scenarios enabled the pharmacist to identify and practice key aspects of teach-back.

Due to the lack of a suitable training video specifically for pharmacists demonstrating teach-back, the primary researcher (EO’M, a TUH pharmacist) created a video which simulated a pharmacist providing teach-back medication counselling to a patient on a high-risk medication (methotrexate) at hospital discharge. Material for the video was adapted from a methotrexate patient information leaflet and the TUH Adult Medicines Guide, with the video transcript provided in Appendix 2. Pharmacists had to watch this 6-min video and were required to read ‘*10 Elements of Competence for Using Teach-back Effectively*’ [11] prior to the intervention. The study team created a standardised procedure for the intervention (Appendix 3), which pharmacists had to read prior to the intervention. The research pharmacist was available throughout the study to address any queries.

#### Prioritisation criteria

Pharmacists prioritised patients for the intervention using predefined criteria in conjunction with professional judgement. Prioritisation criteria were adapted from the TUH Clinical Pharmacy Services Policy to minimise sampling bias. Pharmacists were instructed to prioritise the following patients for counselling: (i) patients with polypharmacy (≥ 5 medications), (ii) patients with multiple prescription changes, (iii) patients newly prescribed high-risk medications or other specific medications (see Appendix 3), (iv) patients identified as at risk of compliance problems, (v) patients with newly diagnosed conditions requiring complex medication regimens (e.g. Parkinson’s disease, epilepsy), and (vi) patients prescribed new medication devices requiring education on administration technique.

#### Patient eligibility and recruitment

Patients were eligible to be recruited from eight medical specialties using convenience and purposive sampling. Based on similar previous studies and resource constraints, a sample size of 40 patients was planned in advance [3, 29]. No power calculation was performed.

Patients were eligible if they met all the following criteria: (i) had a planned discharge from TUH and provided verbal consent to receive discharge medication counselling and a follow-up telephone call, (ii) aged ≥ 18 years, (iii) spoke fluent English, and (iv) prescribed ≥ 1 new medication or had ≥ 1 medication change documented. Patients were excluded if they met any of the following criteria: (i) discharged before a pharmacist provided discharge medication counselling, (ii) unable or unwilling to receive discharge medication counselling, (iii) had significant impairment in vision, verbal communication, or cognitive function and required assistance with their medication, (iv) did not own a mobile phone or telephone, or (v) previously included in the study.

To enhance patient recruitment, a memo was created for nursing staff two weeks into data collection and displayed at ward level to create awareness of the initiative and describe key intervention details. Additionally, the primary researcher spoke to the nurse managers of relevant wards and requested that pharmacists be informed, where possible, when suitable patients were planned for discharge.

#### Data collection

A data collection form and checklist (Appendix 4) was created and issued to intervention pharmacists. Data collection was undertaken over an 8-week period from March to May 2021. Data collection materials were piloted for one week in March 2021 to identify and address any potential process issues. Following pharmacist feedback, minor changes were made to the materials. Data collected during the pilot were included in the study.

The patient’s discharge prescription and/or medication changes confirmed with the medical team were employed as a reference to complete the ‘*Changes to my Medication List*’ (Appendix 5), which was reviewed to ensure appropriateness from a health literacy perspective [30]. New medication and/or changes made to pre-existing medication were documented on this list, which was intended as a compliance aid to remind patients how and when to take their medication and important side effects to be mindful of.

#### Standardised intervention

Nine pharmacists participated, with an average of 8.7 years’ hospital experience. The primary researcher did not participate in the intervention. The procedure (Appendix 3) and counselling checklist (Appendix 4) were provided to all pharmacists to ensure education provided was consistent and followed best practice [30, 31].

Pharmacists recruited patients and performed the intervention as per the following procedure:

(i)  Identify a suitable patient for counselling following prioritisation and eligibility criteria,
(ii)  Obtain patient’s verbal consent,
(iii)  Communicate with patient’s medical team to determine discharge date and provision of sufficient notice for counselling,
(iv)  Identify a list of discharge medications for counselling (new medications and/or medication changes only),
(v)  Verbal confirmation with team of discharge prescription plan for these medications and document in patient’s medical notes,
(vi)  Complete updated list of prescribed medication in ‘*Changes to my Medication List*’,
(vii)  Obtain written information resources for counselling if appropriate,
(viii)  Following the checklist, counsel the patient using teach-back using the medication list,
(ix)  Utilise teach-back to confirm patient’s understanding of key information,
(x)  Ask and answer any question(s),
(xi)  Provide patient with ‘*Changes to my Medication List*’ to take home, and
(xii)  Document counselling points and pharmacist satisfaction with teach-back in patient’s notes [11].

Only patients discharged from Monday-Friday 08.00-16.00 were included, based on pharmacist availability to perform counselling. Patients were counselled on changes made to their pre-existing medication(s) and/or medication(s) newly initiated during inpatient stay only.

### Intervention evaluation

#### Patient telephone survey

Prior to telephone call commencement, the primary researcher completed a certified course on ‘*Communication Skills for Telephone Consultations*’ endorsed by the Health Service Executive, the national provider of health and social care services.

A telephone survey (Appendix 6) including both close-ended questions and one open-ended question was designed to evaluate the intervention’s impact on patient satisfaction and medication understanding. The survey content was informed by the literature [26, 32] and the study team’s experience. Patients who received counselling and granted verbal consent for follow-up were telephoned by the primary researcher within seven days of discharge. Responses to the open-ended question were documented as near-*verbatim* notes by the primary researcher.

#### Pharmacist survey

A survey was developed to evaluate pharmacists’ experience of performing teach-back discharge medication counselling (Appendix 7). The survey content was informed by the literature [11] and the study team’s experience, and included both close-ended questions and one open-ended question. Intervention pharmacists were emailed a link to the online survey on Microsoft® Forms once the intervention was completed. The survey was voluntary and anonymous.

#### Data analysis

Descriptive statistics were calculated using Microsoft® Excel and IBM® SPSS Statistics Version 28 to report patient demographics and data collection components. Qualitative data from the open-ended survey questions were analysed using thematic analysis by study authors EO’M and KD [33].

## Results

### Patient characteristics

Thirty-two patients participated in this study, with their details provided in Table 1. The three most common discharge diagnoses were pulmonary embolism (*n* = 8), atrial fibrillation (*n* = 5), and cerebral infarction (*n* = 5), with the full list provided in Appendix 8.

**Table 1:** Patient details

Descriptor	*n* (%)
*Gender*	
Female	22 (68.8)
Male	10 (31.3)
Age in years, mean (SD; range): 56.5 (18.3; 19–91)	
*Age categories*	
< 45 years	10 (31.3)
45–64 years	10 (31.3)
≥ 65 years	12 (37.5)
Length of stay in days, mean (SD; range): 10.3 (7.6; 0–34)	
Previous hospital admission within 1 year	11 (34.4)
Co-morbidities, mean (SD; range): 2.7 (2.1; 0–8)	
*Co-morbidities prior to admission,*	
0	5 (15.6)
1	6 (18.8)
2	5 (15.6)
3–4	10 (31.3)
≥ 5	6 (18.8)
Charlson Comorbidity Index, mean (SD; range): 3 (2.4; 0–8)	
*Medical Specialty*	
Gastroenterology	8 (25)
Respiratory	5 (15.6)
Rheumatology	5 (15.6)
Age-related healthcare	4 (12.5)
Endocrinology	4 (12.5)
Acute medical unit	3 (9.4)
Haematology	2 (6.3)
Neurology	1 (3.1)
*Prioritisation criteria*	
High-risk medication	19 (59.4)
Priority education medication	19 (59.4)
Multiple changes to prescription	14 (43.8)
Polypharmacy	7 (21.9)
Medication administration education	7 (21.9)
Professional judgement	5 (15.6)
Other	4 (12.5)
Complex medication regimen	2 (6.3)
Compliance Issues	1 (3.1)
*Medication counselling components discussed with patients*	
Medication Name	31 (96.9)
Dose	31 (96.9)
Frequency	32 (100)
Duration	30 (93.8)
Indication	32 (100)
Side effects	32 (100)
Special Instructions	30 (93.8)
Reason for medication changes	26 (81.3)
*Written Resources provided to patients*	
Changes to my medication list	32 (100)
Anticoagulant card^†^	15 (46.9)
Patient information leaflet for a medication^†^	13 (40.7)
Anticoagulant book^†^	11 (34.4)
Steroid card	5 (15.6)
Stroke booklet	2 (6.3)
Asthma Society of Ireland Respimat^®^ information	1 (3.1)
Epipen^®^ booklet^†^	1 (3.1)
Gabapentin information for menopausal symptoms	1 (3.1)
Latent tuberculosis information from HSE	1 (3.1)
Thrombosis Ireland apixaban information leaflet	1 (3.1)

HSE: Health Service Executive. SD: Standard Deviation, ^†^Resource from the licensed manufacturer

### Discharge medication counselling

Nine pharmacists (median 4 patients/pharmacist) provided counselling on 94 medications (mean 2.94 medications/patient; standard deviation [SD] 2.03; range 1–9). This consisted mostly of new medications (*n* = 79; 84%), with the remainder involving medication changes (*n* = 15; 16%). The mean time spent on counselling was 23.6 min (SD 11.9; range 7–60 min). Two thirds of patients received counselling on antithrombotics. Approximately one third of patients were counselled on cardiovascular medications and one quarter of patients received counselling on gastrointestinal medications. The medications counselled on during the study are shown in Table 2. Five patients (15.6%) had no prior comorbidities and no pre-admission medications; three of these were newly prescribed a high-risk anticoagulant.

**Table 2:** Medications counselled on by pharmacists at discharge

Medication type (Anatomical Therapeutic Chemical Classification)	*n* (% of total patients)
**Antithrombotic agents (B01)**	**22 (68.8%)**
Apixaban (B01AF02)	6
Rivaroxaban (B01AF01)	5
Clopidogrel (B01AC04)	3
Aspirin (B01AC06)	3
Edoxaban (B01AF03)	3
Warfarin (B01AA03)	1
Enoxaparin (B01AB05)	1
**Drugs for peptic ulcer and gastro-oesophageal reflux disease (A02B)**	**8 (25%)**
Pantoprazole (A02BC02)	5
Omeprazole (A02BC01)	1
Lansoprazole (A02BC03)	1
Sucralfate (A02BX02)	1
**Beta blocking agents (C07)**	**7 (21.9%)**
Bisoprolol (C07AB07)	5
Metoprolol (C07AB02)	1
Nebivolol (C07AB12)	1
**Calcium Channel Blockers (C08)**	**5 (15.6%)**
Lercanidipine (C08CA13)	3
Amlodipine (C08CA01)	2
**ACE inhibitors (C09A)**	**5 (15.6%)**
Ramipril (C09AA05)	4
Perindopril (C09AA04)	1
**Vitamin Preparations (A11)**	**4 (12.5%)**
Multivitamin (A11AA03)	1
Vitamin B Complex (A11BA)	1
Thiamine (A11DA)	1
Pyridoxine (A11HA02)	1
**Blood Glucose Lowering Drugs (A10B)**	**4 (12.5%)**
Gliclazide (A10BB09)	2
Metformin (A10BA02)	1
Sitagliptin (A10BH01)	1
**Diuretics (C03)**	**3 (9.4%)**
Bumetanide (CO3CA02)	2
Spironolactone (C03DA01)	1
**Drugs for obstructive airway diseases (R03)**	**3 (9.4%)**
Tiotropium bromide (R03BB04)	1
Vilanterol, umeclidinium bromide, and fluticasone furoate (R03AL08)	1
Formoterol and budesonide (R03AK07)	1
**Antiepileptics (N03)**	**3 (9.4%)**
Sodium valproate (N03AG01)	1
Gabapentin (N03AX12)	1
Levetiracetam (N03AX14)	1
**Lipid Modifying Agents (C10)**	**3 (9.4%)**
Atorvastatin (C10AA05)	3
**Corticosteroids (systemic) (H02)**	**3 (9.4%)**
Prednisolone (H02AB06)	3
**Antibacterials for systemic use (J01)**	**3 (9.4%)**
Amoxicillin (J01CA04)	2
Co-trimoxazole (J01EE01)	1
**Antihypertensives (C02)**	**2 (6.3%)**
Doxazosin (C02CA04)	2
**Antipsychotics (N05)**	**2 (6.3%)**
Levomepromazine (N05AA02)	1
Quetiapine (N05AH04)	1
**Immunosuppressants (L04)**	**2 (6.3%)**
Fingolimod (L04AA27)	1
Methotrexate (L04AX03)	1
**Other**	**15 (46.9%)**

### Patient telephone survey

All patients were successfully contacted by telephone within 7 days of discharge (mean 3.16 days; SD 1.6; range 1–7 days) and answered all closed-ended questions. Overall, 93.7% (*n* = 30) of patients were satisfied with the counselling experience and the information they received from the pharmacist in hospital. Responses to the remaining closed-ended survey statements are provided in Table 3. Twenty-eight patients (87.5%) provided comments about the counselling; the themes generated from these comments are provided in Table 4. The two patients (6.3%) who were dissatisfied (Patient 18 and 21) with the experience were counselled by different pharmacists, and both provided comments on the final question. Additional details on this are provided in Appendix 9.

**Table 3:** Patient responses to closed-ended survey statements

Statement	Agree	Undecided	Disagree
It is important to get information about changes to my medications before I leave hospital	100% (*n* = 32)	-	-
It is important to get information about possible side effects of my medications before I leave hospital	96.9% (*n* = 31)	-	3.1% (*n* = 1)
Before I went home, the pharmacist spent enough time with me explaining my medications	87.6% (*n* = 28)	6.3% (*n* = 2)	6.3% (*n* = 2)
The pharmacist explained the purpose of my medications, in a way which I could understand	93.7% (*n* = 30)	3.1% (*n* = 1)	3.1% (*n* = 1)
The pharmacist explained how and when to take my medications at home, in a way which I could understand	93.7% (*n* = 30)	3.1% (*n* = 1)	3.1% (*n* = 1)
Possible side effects of my medications to watch out for when I went home were explained, in a way which I could understand	90.6% (*n* = 29)	-	9.4% (*n* = 3)
Changes to my medications were explained to me in a way which I could understand	93.7% (*n* = 30)	3.1% (*n* = 1)	3.1% (*n* = 1)
The written or printed information I received in hospital (e.g., ‘*Changes to my Medication list*’) helped me to understand my medications when I went home	93.7% (*n* = 30)	-	6.3% (*n* = 2)
I am more confident about my knowledge of my medication after my discussion with the pharmacist in hospital	93.7% (*n* = 30)	-	6.3% (*n* = 2)

**Table 4:** Patient Feedback about Discharge Medication Counselling

Theme	Comments
*1. Clarity and understanding*	
Patients valued the information and clarity of explanation, which improved their understanding of medication use, indication, and side effects This helped reduce previous confusion, and provided reassurance and confidence to manage medication Some, however, still did not understand, even with the teach-back counselling	“*I was given crystal clear instructions on how to take my new tablet… I found the advice given to me in hospital just brilliant…”* [Patient 11] “*Education is amazing for patients…”* [Patient 23] *“I understand all my medicines that I take at home now”* [Patient 17] *“…it was the first time I knew what my tablets were all about.”* [Patient 9] “*When I go home from hospital, I feel it is very important for me to be aware of the medication side effects and what medication interacts…”* [Patient 12] *“…made me aware of the reason I was put on Eltroxin®; I did not know was it for under- or over-active thyroid. I was confused until I was told*.” [Patient 10] *“I am confident to manage my tablets at home”* [Patient 23] *“ …The pharmacist put my mind at rest…”* [Patient 27] *“…needed to explain the information a bit more.”* [Patient 18] “*…I did not know that I had to take new tablets… I should have been told…”* [Patient 21]
*2. Information provision before discharge*	
The importance of medication counselling before discharge was noted; some said it should be provided to all patients. The time provided was not sufficient for everyone, with community pharmacist involvement mentioned	“*This is a great idea. I wish I could get all this information every time I visit the hospital.”* [Patient 6] “*People should be spoken to every time in hospital and explained why their tablets have changed.”* [Patient 9] “*When I went home to my chemist, I was not confused. I was able to tell them about the change to my Rx.”* [Patient 11] “*…I needed more time to be told about my tablets…my local chemist had to explain again…”* [Patient 18] “*I am very happy with the time taken to advise me on my medication.*” [Patient 22]
Too close to discharge may not be the most suitable time for counselling as patients may be unwell or distracted, and may find the information excessive	“*Very rushed as I was waiting to be discharged (porter and my husband waiting for me). Therefore, I did not take in information and could not concentrate on what pharmacist was saying. I suggest to get advice about medicines earlier during stay and not at discharge.*” [Patient 7] “*I can't remember much of the pharmacist talking to me as I was a bit upside down in hospital…”* [Patient 20] “*I don't think it is important for me to learn about side effects of my tablets. This is too much information to take in…” *[Patient 28]
Written information was easy to understand and supported the verbal counselling. It was useful as a point of reference at home, especially with the possibility of forgetting	“*The list of my new medication was very clear and easy to follow…”* [Patient 22] “*Excellent documentation helped me to understand new medication*.” [Patient 4]
	“*The form acted as a reminder how to take my tablets when I went home. I think everybody should get written information…”* [Patient 5]
	“*The warfarin booklet is very useful to refer to*.” [Patient 27] If I received no written sheet, I would be very confused. [Patient 6]
	“*…I did not refer to the leaflet, as I felt I knew enough about my tablets…”* [Patient 7]
*3. Pharmacist approach and performance*	
Patients were very satisfied with the service overall. They appreciated the value of teach-back and repeating instructions, and that the pharmacists took time to ensure understanding and answer any questions where needed	“*Very happy with the service.*” **[Patient 2]** “*I am 100% satisfied with the advice”.* [Patient 6] “*…it was a "brilliant experience.*" [Patient 10]
	“*…helped me to understand in a clever way, by checking and asking me to tell her in my own way…”* [Patient 11]
	“*…made sure to check a number of times that I understood the information explained to me about my new medicine*.*”* [Patient 15]
	*“I appreciate the time taken to explain my new medication”.* [Patient 12] *“The advice given was not rushed.”* [Patient 9]
	“*The pharmacist was excellent; she answered all my questions…”* [Patient 22] *“I liked that I could ask questions without feeling stupid…”* [Patient 27]
	*“I was made feel very important and the pharmacist had great respect for me as a patient…”* [Patient 9]
Pharmacists were perceived as professional, helpful, friendly, and easy to talk to – but the importance of not using jargon was noted	“The pharmacist was *very professional, very helpful, clear, and brilliant at her job…”* [Patient 8] “*…easy to talk to, she had a friendly manner.”* [Patient 13]
	*“She explained my new medication in layman's terms. I am very happy with the information as I never took a tablet in my life.”* [Patient 8]
	*“She used jargon that professionals speak that I could not understand.”* [Patient 18]

### Pharmacist survey

Eight of the nine (88.8%) pharmacists completed the anonymous survey to provide feedback on the intervention within 2 weeks of data collection completion. All respondents agreed that i) they received adequate education and training to provide the teach-back method for counselling, and ii) the teach-back method is feasible to apply in clinical practice, and iii) teach-back is an important and effective communication method to help pharmacists ensure patients understand their medication. Whilst most pharmacists (*n* = 6; 75%) agreed that they were confident to use teach-back to undertake medication counselling and intend to adopt this approach in future practice, two respondents (25%) answered ‘*Neither Agree nor Disagree*’. All pharmacists provided a response in the open text box, where they were asked to include any limitations identified and suggestions to improve the discharge medication counselling process. The themes generated from these comments, with supporting quotations, are provided in Table 5.

**Table 5:** Pharmacist Feedback about Discharge Medication Counselling

Theme	Comments
*1. Implications for patient care*	
Potential to improve patients’ adherence, knowledge, and confidence with medication	“*It was apparent that my counselled patients felt that the information provided was beneficial and they seemed more confident / knowledgeable on their medicines on discharge.*” **[Pharmacist 4]**
	“*It gives the opportunity to pharmacists to make a real difference around adherence…”* **[Pharmacist 3]**
Patient gratitude with intervention	“*Feedback received from patients was mostly positive… There were obvious gaps in her knowledge, even after applying teach-back.”* **[Pharmacist 5]**
	“*I found the people I educated to be so very grateful for the information, and I believe that the education will have a positive impact on their compliance and adherence in the future*.” **[Pharmacist 2]**
Effective counselling method, and highlighted patients’ need for discharge counselling	*“…was more of an effective method of counselling than current practice…”* **[Pharmacist 6]**
	“*This project proves there is a need for patient counselling on discharge…”* **[Pharmacist 5]**
Some patients found it challenging	“*The teach-back method seems useful, but for those patients who could not relay the information back it was very challenging.*” **[Pharmacist 3]**
Focus distracted by imminent discharge	*“On one occasion, the patient’s NOK was downstairs waiting – it’s questionable as to how much the patient actually paid attention to the counselling session as she was very eager to leave.”* **[Pharmacist 5]**
*2. Impact on pharmacist practice*	
Beneficial for pharmacists: learning new technique; increased job satisfaction; increased patient contact; highlights pharmacist's role	*“It’s a great experience for pharmacists to learn a new counselling technique…”* **[Pharmacist 8]**
	“*I didn't expect that discharge counselling would increase my job satisfaction as a pharmacist; while some patients clearly benefit more than others, I feel that it is an important aspect of our role.*” **[Pharmacist 4]**
	“…*I really enjoyed the additional contact time with patients… I do believe these are very worthwhile and gratifying activities for pharmacists… and also makes patients actually 'see' our input as opposed to always being invisible in the background!”* **[Pharmacist 3]**
Need to focus on medication counselling in future practice balanced against workload	*“I love the overall idea of using the teach-back method of communication… However, this is not very feasible with our workload at present… I was always stressed about not being on top of workload… …more focus is needed from the pharmacy side on counselling, as it is a crucial area for us as medical professionals, and would often help prevent readmissions or medical issues happening in the future for patients.*” **[Pharmacist 2]**
*3. Environmental challenges*	
Time constraints, busy workload, and need for additional staffing for intervention	“*…very time consuming getting leaflets together for discharge counselling…*” **[Pharmacist 1]**
	“*…undertaking discharge is time consuming and would require additional staffing*.”** [Pharmacist 4]**
	“*Time constraints was another limitation, a heavy patient workload in addition to other responsibilities made it difficult to complete the tasks required.”* **[Pharmacist 5]**
	“*The main limitation is time. It would be amazing to be in a position to counsel every patient on new medication on discharge but we don't currently have the time to do this for all patients, only high-alert medications such as anticoagulants*.” **[Pharmacist 8]**
Need for finalised discharge medication plan and clear communication on this	*“…medical team at times do not communicate effectively when patients are ready for discharge and discharge medications are not finalised for the pharmacists to know what to counsel on.…” ***[Pharmacist 7]**
	*“…counselling a patient without a discharge prescription in hand is inherently a risky process—diuretic doses often change last minute in CCF patients; decisions about whether to restart medications (e.g. antihypertensives withheld during an in-patient stay) will often be made at the last minute.”* **[Pharmacist 4]**
	“*Patients were counselled immediately before discharge to ensure no further changes to the medications occurred*.” **[Pharmacist 5]**
Discharge timing: patients discharged outside pharmacist working hours	“*Great initiative but tricky to implement in practice. Found lots of patients discharged after pharmacist working hours and decisions still to be made about discharge medications meant that it was not feasible to counsel without concrete plan.”* **[Pharmacist 1]**
Difficulties in recruiting eligible patients or in completing the intervention with those eligible	“*Locating patients posed problematic also. On numerous occasions, patients were enlisted into the project but due to unforeseen circumstances their discharge was postponed. This made the data invalid.”* **[Pharmacist 5]**
*4. Suggestions for improvement*	
Need for discharge medication reconciliation	“*…discharge counselling cannot be done fully without some element of pharmacist discharge medication reconciliation. In my view, both are quite interlinked unless counselling on just one medicine in isolation, but this is difficult as naturally enough patients often ask about other medications.*” **[Pharmacist 3]**
Pharmacist aiding with discharge prescription	“*If pharmacists were undertaking medication reconciliation on discharge and / or generating the discharge prescription plus counselling as part of the discharge process, some of these issues may be ironed out.”* **[Pharmacist 4]**
Having patient carer/relative present	“*It may be beneficial to have NOK/carer *etc. *present at the counselling when COVID restrictions are lifted.*” **[Pharmacist 3]**
Availability of generic counselling information Partnership with community pharmacists	“*…risk of patient getting different brand on discharge and causing confusion. Wonder is it better to have generic information available or perhaps look at engaging community pharmacists on discharge counselling.”* **[Pharmacist 1]**
Synchronous/in-person pharmacist training	“*A 'live' training might better upskill pharmacists on how to apply the teach-back method… I understand this was not possible with COVID.”* **[Pharmacist 3]**

CCF: Congestive cardiac failure; NOK: next-of-kin

## Discussion

### Statement of key findings

This study describes innovative research on the development and evaluation of a pharmacist-provided discharge medication counselling procedure using teach-back in an acute university hospital. A standardised approach to counselling was adopted, which incorporated prioritisation criteria to target patients that may benefit most from pharmacist counselling at discharge. There has been limited literature to date reporting pharmacists utilising teach-back globally [7], with inconclusive evidence to show that teach-back should be recommended as a universal approach for pharmacist-patient medication counselling. Notably, this is the first study of its kind to evaluate the patient’s perspective on teach-back medication counselling by pharmacists. This initiative was shown to be feasible to use at discharge in a busy hospital setting and accepted by both pharmacists and patients. Importantly, teach-back had a positive effect on patient outcomes; most patients self-reported that they understood the information explained to them by the pharmacist and were satisfied with the counselling.

### Strengths and weaknesses

The study findings are limited by the small sample size (due to staff shortages during the COVID-19 pandemic) and its single-centred nature; however, the included patients were diverse in terms of age, comorbidities, and discharge diagnosis; therefore, this might suggest that the results may be generalisable to a variety of patient cohorts. Whilst this study lacked a control group to ascertain the intervention’s true impact on patient outcomes, Jager et al*.* have reported that patients who received teach-back education were more likely to have increased satisfaction compared to standard care [34]. No validated tools were utilised to measure patient satisfaction and medication understanding. Consideration was given to validated tools such as the Satisfaction about Information with Medicines Scale and the Medication Understanding Questionnaire. However, both tools were deemed unsuitable for this study due to the considerable time required to complete over the telephone to assess study outcomes [35, 36]. Patients were not formally assessed on the recall of correct responses to teach-back questions. Furthermore, the patient survey may have introduced social desirability bias – possibly feeling more obliged to report greater satisfaction than they actually felt.

This study’s pharmacists were trained on teach-back utilising an evidence-based educational toolkit endorsed by international healthcare institutions [11]. To the authors’ knowledge, this is the only study to date that has described in detail the pharmacist training on teach-back utilising an evidence-based resource. It was not possible to conduct in-person training on teach-back due to social distancing with COVID-19; this may have affected intervention fidelity and its intended translation into practice. While response bias is possible in the pharmacist survey, the likelihood is minimised by the fact that respondents provided both negative and positive comments.

### Interpretation and implications for future research and practice

Similar teach-back studies have described pharmacists implementing a variety of interventions for medication counselling, demonstrating that there is currently no standardised evidence-based teach-back approach for pharmacist-patient education [13–22, 37, 38]. A scoping review of discharge medication counselling by Bonetti et al*.* identified a limited uptake of teach-back by pharmacists [7]. Consequently, this study addressed a research gap by training pharmacists on teach-back utilising an evidence-based resource [11] and developing a standardised checklist for discharge medication counselling. This process incorporating teach-back involves many steps and checklists help ensure critical steps of a process are not missed, improve consistency of care, and enhance patient outcomes [39]. Previous studies where pharmacists incorporated checklists to deliver teach-back improved inhaler technique and patient satisfaction [13–16]. This study further demonstrates the value in standardising complex multicomponent processes utilising written checklists to achieve implementation fidelity and in replicating consistent teach-back counselling in pharmacy practice [30, 39].

As the educational toolkit provided was intended for HCPs in general, it was endeavoured to adopt an evidence-based pharmacist-patient communication strategy as a training method that incorporated a role play session to practice teach-back [40]. However, this was not possible due to the COVID-19 pandemic implications of social distancing and pharmacist time constraints. Alternatively, a teach-back medication counselling video was created for pharmacists which demonstrated the correct utilisation of teach-back. All pharmacists agreed the teach-back approach was feasible to incorporate into practice and was an effective communication method to help pharmacists ensure patients understand their medication. This is corroborated as most (93.7%) patients self-reported they understood the medication counselling. This finding is important as medication counselling is a futile exercise unless patients can demonstrate correct teach-back of key information.

The Bonetti et al*.* scoping review found that only half the studies from the literature reported counselling time, whereby the average time spent by pharmacists performing teach-back medication counselling ranged from approximately 5–52 min [15–17, 22, 38]. The average time spent by pharmacists in this study was 23.6 min (range 7–60 min); whilst this is less than the median 30 min reported by Bonetti et al*.* (which was not focused solely on teach-back counselling) [7], most patients (87.6%) agreed this amount of time was sufficient. Reduced counselling time in the present study may be explained as pharmacists focused on new medications and/or medication changes only. However, this did not account for time spent identifying patients, confirming medications with the patient’s medical team, and preparing documentation prior to counselling. Therefore, the entire process may have taken considerably longer. For example, one study reported that pharmacists took an average of 52 min to prepare and counsel a patient using teach-back [20]. More importantly, wide variability in counselling time significantly hinders the practicality of routinely providing pharmacist-conducted teach-back medication counselling at discharge; therefore, further research is required to investigate the cost-effectiveness considering the patient benefits alongside the resources required. Prioritisation criteria, as were used in this study, may be an integral component in providing an initiative like this with limited resources [20].

Remarkably, patients do not understand or are unable to remember approximately half of the medical information communicated by HCPs [10]. Moreover, a German study confirmed that less than half (43%) of patients understood a standardised written medication plan [41]. This finding suggests that written information alone is not sufficient to ensure correct patient medication administration and verbal instructions are required to reinforce this. Therefore, along with teach-back in this study, all patients were provided with a ‘*Changes to my Medication List*’ as written information for referral at home. Bonetti et al*.* found that pharmacists performing medication counselling rarely (15.8%) provided a written medication list to patients [7], even though a Cochrane review has shown that a combination of both written and verbal elements standardised information for patients at hospital discharge, improved information recall, and increased patient knowledge and satisfaction [42]. Similarly, findings from the present study align to this as most (93.7%) patients believed that the written resources provided supported medication understanding. Future studies should undertake independent assessment of patient outcomes using validated tools to minimise self-reporting bias. Ideally, a formal assessment of teach-back is necessary to ensure a comprehensive evaluation of medication understanding.

One of the main drivers of this initiative was the results from the 2019 National Inpatient Experience Survey, which showed that patient scores from the study site regarding information about medication side effects at discharge were below the national average [26]. Bonetti et al*.* reported that only just over half (56.6%) of medication counselling studies internationally reported pharmacists discussing adverse drug reactions with patients at discharge [7]. Most patients in this study (90.6%) agreed that possible side effects were explained to them in a way they could understand, supporting the previous evidence showing teach-back as an effective communication method for medication side effect counselling by pharmacists in hospital, with increased patient satisfaction [18]. In an era of increasing hospital transparency, patient satisfaction is a key area of quality patient care to address. Most patients in this study (93.7%) were satisfied with the counselling and with the medication information provided. This patient satisfaction score is comparable to other studies that reported patient satisfaction with pharmacist-delivered teach-back medication counselling – 94% [13, 19] and 91% [16] respectively.

It must be highlighted that some pharmacists and patients in this study noted that discharge may not be the most suitable time for medication counselling. The busy environment may cause distractions and negatively affect patients’ knowledge retention. The pharmacists identified several other barriers to discharge medication counselling such as limited resources, time constraints, insufficient communication about patient discharge, and pharmacist working hours. These barriers are consistent with findings from a Canadian study on a similar intervention [8]. Furthermore, pharmacists emphasised that the absence of discharge medication reconciliation was a limitation. Staff shortages and time constraints meant pharmacists only counselled patients on medication changes and/or new medications. Although the impact of pharmacist-provided medication reconciliation and counselling at discharge on health outcomes and medication errors has been inconsistent [21, 22, 37], discharge medication reconciliation is advocated as evidence-based standard practice [2, 9].

In 2021 in the United Kingdom, a new discharge medication referral service was implemented to improve transfer of care; however, its impact is yet to be established [43]. A hospital refers a patient to their community pharmacy and electronically transfers the patient’s discharge medication information. The community pharmacist conducts medication reconciliation, counsels patients on discharge medications, and ensures the patient understands medication changes. Perhaps a similar initiative, as suggested in this study’s results, may be a more practical solution given the discharge process challenges that currently exist in Irish hospitals. Another suggestion to increase discharge medication counselling would be an electronic alert sent to pharmacists to flag patients’ imminent discharge or highlight patients for priority counselling.

## Conclusion

This study has described the development and evaluation of a standardised discharge medication counselling procedure by pharmacists utilising teach-back in an acute teaching hospital. Overall, 93.7% of patients were satisfied with the counselling and with the information provided, with the same proportion stating increased confidence regarding medication knowledge. Whilst this study adds to the growing evidence supporting teach-back as an effective communication method for discharge medication counselling, future larger-scale multi-centre randomised controlled trials are required to determine the true impact of pharmacist teach-back interventions on patient satisfaction, medication understanding and adherence, and the associated cost-effectiveness. Although this intervention was feasible to utilise and was acceptable both to pharmacists and patients, time constraints in busy hospital settings and the need for accompanying medication reconciliation pose key considerations before implementing interventions like this going forward. Ultimately, the comprehensive description of the intervention components and evidence-based training from this study should help inform the development of discharge medication counselling services going forward.

## Supplementary Information

Below is the link to the electronic supplementary material.

Supplementary file1 (DOCX 87 kb)
