# Supplementary material for: Development and evaluation of pharmacist-provided teach-back medication counselling at hospital discharge
Source: Int J Clin Pharm. 2023 Apr 24;45(3):698–711. doi: 10.1007/s11096-023-01558-0 (PMC10124684; doi:10.1007/s11096-023-01558-0)
Supplement: Supplementary file 1 — Supplementary file1 (DOCX 87 kb) [file 11096_2023_1558_MOESM1_ESM.docx]

**Appendix 1**

**The TIDieR (Template for Intervention Description and Replication) Checklist:**

| **Item number** | **Item** | **Where located in paper** |
| --- | --- | --- |
|  | **BRIEF NAME** |  |
| **1.** | Provide the name or a phrase that describes the intervention. | Title; Methods (Study design, Standardised intervention). |
|  | **WHY** |  |
| **2.** | Describe any rationale, theory, or goal of the elements essential to the intervention. | Introduction; Methods (Pharmacist training on intervention; Prioritisation Criteria; Data Collection; Standardised intervention). |
|  | **WHAT** |  |
| **3.** | Materials: Describe any physical or informational materials used in the intervention, including those provided to participants or used in intervention delivery or in training of intervention providers. Provide information on where the materials can be accessed (e.g. online appendix, URL). | Methods (Pharmacist training on intervention; Data Collection; Standardised intervention). |
| **4.** | Procedures: Describe each of the procedures, activities, and/or processes used in the intervention, including any enabling or support activities. | Methods (Pharmacist training on intervention; Prioritisation Criteria; Patient eligibility and recruitment; Data Collection; Standardised intervention). |
|  | **WHO PROVIDED** |  |
| **5.** | For each category of intervention provider (e.g. psychologist, nursing assistant), describe their expertise, background and any specific training given. | Methods (Pharmacist training on intervention; Standardised intervention). |
|  | **HOW** |  |
| **6.** | Describe the modes of delivery (e.g. face-to-face or by some other mechanism, such as internet or telephone) of the intervention and whether it was provided individually or in a group. | Methods (Standardised intervention). |
|  | **WHERE** |  |
| **7.** | Describe the type(s) of location(s) where the intervention occurred, including any necessary infrastructure or relevant features. | Methods (referred to throughout that this was conducted with patients in hospital prior to discharge). |
|  | **WHEN and HOW MUCH** |  |
| **8.** | Describe the number of times the intervention was delivered and over what period of time including the number of sessions, their schedule, and their duration, intensity or dose. | Methods (Standardised intervention). The mean counselling time is recorded in the Results section. |
|  | **TAILORING** |  |
| **9.** | If the intervention was planned to be personalised, titrated or adapted, then describe what, why, when, and how. | Not applicable. |
|  | **MODIFICATIONS** |  |
| **10.** | If the intervention was modified during the course of the study, describe the changes (what, why, when, and how). | Not applicable. |
|  | **HOW WELL** |  |
| **11.** | Planned: If intervention adherence or fidelity was assessed, describe how and by whom, and if any strategies were used to maintain or improve fidelity, describe them. | Not applicable. |
| **12.** | Actual: If intervention adherence or fidelity was assessed, describe the extent to which the intervention was delivered as planned. | Not applicable. |

**Appendix 2**

**Background Video Information and Transcript for Discharge Patient Medication Counselling Video using the Teach-back Method.**

**Background to video:**

The patient’s name is Monika, a 35-year-old female newly diagnosed with rheumatoid arthritis. She is to be discharged from hospital on a new medication called methotrexate.

**The pharmacist (Emer) has been asked to:**

- Counsel the patient on methotrexate
- Ensure the patient knows the following:
  - The name of the new medicine.
  - The reason the medicine has been prescribed.
  - Dose and frequency of administration.
  - Side effects and warning signs that the patient is to watch out for.
  - Importance of monitoring.
  - Medications to avoid.
  - Contraceptive advice.
- Use clear and simple language and ask open-ended questions using the teach-back technique to ensure the patient understands the information provided.

**Transcript**

**Pharmacist:** *My name is Emer. I am a pharmacist that works in the hospital. I would like to have a chat with you before you go home to explain your new medication if that’s okay?*

**Patient*:*** *Yes, I am happy to find out about my new tablet.*

**Pharmacist:** *Monika, you have been started on a new tablet called methotrexate to help control your arthritis. Methotrexate slows down your body’s immune system and helps reduce inflammation in your joints. You need to take 4 tablets (10mg) once a week, on the same day each week, e.g. every Monday. It is very important that you only take methotrexate once a week. Taking too much methotrexate (e.g. every day) can cause serious effects and may be fatal. You will need to attend your doctor regularly for blood tests to ensure your tablet is controlling your arthritis and you are not experiencing severe side effects. Swallow the tablets whole with a drink of water. You can take your tablets before or after food. Wash your hands after touching the tablets.*

*Like all medicines, methotrexate can cause common side effects, although not everyone gets them (e.g. stomach upset, loss of appetite, headache). It may make you feel tired and dizzy. You should not drive or use machines when you first start to take this medicine until you are certain that you are not getting these side effects. Serious side effects are very rare, but I just want to let you know about them. Tell your doctor straight away if you develop:*

- *Sore throat or an infection*
- *Fever or chills*
- *Mouth ulcers*
- *Easy bruising or bleeding*
- *Breathlessness*
- *Dry cough*
- *Unexplained rash*

*It is very important to tell your doctor, dentist, or pharmacist that you are taking methotrexate. Always check that over-the-counter medicines are suitable to use with methotrexate before taking. There are many medicines that affect the way methotrexate works. Avoid aspirin and anti-inflammatory painkillers like ibuprofen.*

*Finally, I would like to mention that is important that you do not get pregnant while taking methotrexate, as this medicine can harm an unborn baby. Speak to your doctor if you plan to have children in the future. You must ensure to use an effective method of contraception* *during treatment and for at least 6 months after stopping methotrexate.*

*I have provided you with a lot of information today and I want to make sure I explained everything clearly. Can you tell me in your own words how you are going to take methotrexate when you go home?*

**Patient:** *Okay. Well, I have to take 10 tablets once a week every Monday for my arthritis. I must wash my hands after touching the tablets.*

**Pharmacist:** *Yes you are right in saying about washing your hands. But I am going to go over again how much methotrexate you need to take every Monday. You will take 4 tablets once a week. FOUR tablets make up your weekly dose of 10mg. You do not take 10 tablets. That is too much. You just take 4 tablets together on a Monday.*

*So, can you tell me again how much methotrexate you are going to take every week?*

**Patient:** *Okay, I got a bit confused. So, I just take 4 tablets together once a week, which gives me the 10mg dose?*

**Pharmacist:** *Yes Monika, that is correct. You just take 4 tablets together every Monday.*

*I have given you lots of information about important side effects to watch out for. Can you tell me about two* *serious side effects and what should you do if you experience any?*

**Patient:** *You told me to watch out for any infection or sore throat and any chest problems like a dry cough or difficulty breathing. I need to contact my doctor straight away if I get any of these side effects.*

**Pharmacist:** *Yes Monika, that is correct. You can find a list of serious side effects to watch out for in the patient information leaflet provided with your medicine. If you are ever unsure, contact your doctor.*

*I would like to finish up by asking what questions do you have?*

**Patient:** *You told me not to take anti-inflammatory pain killers. What can I take if I get a headache?*

**Pharmacist:** *Paracetamol is a safe option to take for a headache instead of anti-inflammatory pain killers.*

**Patient:** *Also, I am wondering can I have a few drinks while I am taking my new tablet?*

**Pharmacist:** *There is no data on the safe use of alcohol with methotrexate. Due to the possible effects on your liver, it is best to limit your alcohol intake to 4 units per week.*

**Patient:** *Okay, that’s good to know. Thanks for your advice today*.

**Pharmacist:** *It was my pleasure. You are more than welcome.*

**Appendix 3**

**Procedure for Pharmacist Discharge Medication Counselling**

1. Identify target patients early during inpatient stay following admission medication reconciliation. Determine the planned discharge date if possible.
2. Pharmacists should prioritise the following patients in conjunction with professional judgement for discharge medication counselling:

- Polypharmacy: patients prescribed at least 5 or more medications.
- Patients with multiple changes made to their prescription.
- Patients newly prescribed high-risk medications, e.g. anticoagulants, digoxin, methotrexate, opioids, gliclazide, paracetamol products.
- Patients newly prescribed the following medications: amiodarone, carbimazole, steroids (high dose/long term), tuberculosis medications, High-tech medications, exempt medicinal products.
- Patients identified as at risk of compliance problems.
- Patients with newly diagnosed conditions requiring complex medication regimens, e.g. Parkinson’s disease, epilepsy.
- Patients prescribed new medication devices requiring education on administration technique, e.g. an inhaler.

1. The following patients are to be **included** in the study:

- Patients that have planned discharge from Tallaght University Hospital (TUH).
- Patients that provide verbal consent to receive discharge medication counselling and follow-up telephone call.
- Patients that speak fluent English.
- Patients aged 18 years or older.
- Patients that are prescribed at least one new medication or have at least one medication change documented.

The following patients are to be **excluded** from the study:

- Patients that are discharged before pharmacist provided discharge medication counselling.
- Patients unable or unwilling to receive discharge medication counselling.
- Patients with significant impairment in vision, verbal communication, or cognitive function requiring assistance with their medication.
- Patients that do not own a mobile phone or telephone
- Patients previously included in the study.

1. Ask patient for verbal **consent** prior to discharge medication counselling, preferably during or after admission medication reconciliation. Ensure permission is granted to contact patient by telephone after discharge.
2. Communicate with patient’s medical/surgical team to provide sufficient notice before discharge for patient education. Alternatively, the pharmacist can monitor the patient’s planned discharge date.
3. Identify a discharge medication list of new medications and/or medication changes only. **The patient’s full discharge medication list is not required**. Highlight any unresolved issues with the medical/surgical team. Confirm medication changes verbally with team and document on medical notes.
4. Document new medications and or medication changes on the ‘changes to my medication list’ template provided. Pharmacists will use this resource as a counselling tool. In addition, please document the following for each medication on the list:

- Medication name and strength, medication frequency, medication indication, medication side effects, medication special instructions.

1. Make a copy of ‘Changes to my Medication List’ for the Research Pharmacist.
2. Obtain **written information resources** for patient counselling if appropriate, e.g. anticoagulant alert card, patient information leaflet (PIL) for medication or community drug schemes. PILs can be found on the TUH Medical Photography intranet page.
3. Communicate with patient using the **teach-back** method. Follow the ‘10 Elements of Competence for Using Teach-back Effectively’. Introduce yourself to the patient and explain that ‘*I am going to have a chat with you to ensure you understand your new medication and/or changes made to your prescription while in hospital*.’
4. Review the ‘Changes to my Medication List’ with the patient**. Counsel** patient on new medications and medication changes. Provide patient counselling on the following components for each medication:

- Drug name, dose, frequency of administration, indication, duration, common or significant side effects, special instructions (e.g. with/after food), medication change and reason (e.g. new, dose increase/decrease).
- Advise patient if a pre-admission medication has been stopped or replaced by another medication.

Refer to PILs available from the Health Products Regulatory Authority (HPRA) if necessary.

1. Ask the patient the following question to evaluate comprehension of counselling provided: *‘I have provided you with a lot of information today, and I want to make sure I explained everything clearly. Can you tell me in your own words how you are going to take your medication when you go home?’*
2. If needed, clarify and tailor counselling, check patient recall and understanding again; repeat this process until full comprehension is achieved.
3. Thank the patient for their time and close the counselling session by asking the patient: ***‘What questions do you have?’***. Do **not** ask patient ‘*have you any questions?’*
4. Inform patient they will receive a follow-up telephone call in the next week where another pharmacist will check to see how they are getting on with their medications*.* Ask for patient’s telephone number and a convenient time to call. Document on data collection form.
5. Provide patient with the ‘Changes to my Medication List’ to take home.
6. Document patient medication counselling points provided in the **patient’s medical notes**, i.e. medication(s) counselled, counselling components, written information resources provided, patient’s question(s) addressed, and pharmacist satisfaction with patient understanding of information provided.
7. Ensure data collection form and checklist (double sided) and changes to my medication list are complete. Scan and email same to the Research Pharmacist .

**Appendix 4 Data Collection Form and Checklist for Pharmacist Discharge Medication Counselling**

Place Patient ID Sticker below Study ID: _______

Name MRN

DOB dd/mm/yyyy

M/F

**Age:** _________ **Pharmacist:** ___________________________________

**Speciality:** ____________________ **Date of data collection:** _ dd/mm/yyyy_________________

**Admission date:** _dd/mm/yyyy/mm/yyyy_______________

**Discharge date:** ______________ **Total (days):**_______

**Date of last admission:** __________________________

| **Medication Counselling** | |
| --- | --- |
| **Drug name** | **New (N),**  **Change in dose and/or**  **frequency/medication held or stopped (C)** |
|  |  |
|  |  |
|  |  |
|  |  |
|  |  |
|  |  |
|  |  |
|  |  |

**Counselling time:**  **Patient telephone number:** _____________________________ **Start:** ____________

**Stop:** ____________ **Alternative telephone number:** __________________________

**Total (mins):** _______ **Convenient time to call:** ________________________________

**Reason for Admission/New Diagnosis:** _______________________________________________________________________________________

_______________________________________________________________________________________

**Past Medical History (PMH)/Co-morbidities:**

_______________________________________________________________________________________

_______________________________________________________________________________________

_______________________________________________________________________________________

**After Counselling**

**During Counselling**

**Before Counselling**

Name MRN

DOB dd/mm/yyyy

M/F

| **17. Document patient medication counselling points in medical notes.** 🞎  🞎 Document if pharmacist satisfied with patient understanding of information provided. |
| --- |
| **18. Ensure the following documentation are complete:**  🞎 data collection form and checklist for pharmacist discharge medication counselling (two-sided)  🞎 changes to my medication list |
| **Scan and email the above documentation to Research Pharmacist.**  🞎 |

| **10.**  Communicate using the teach-back method. Follow the ‘10 Elements of Competence for Using Teach-back Effectively’:   - Use a caring tone of voice and attitude. - Display comfortable body language and make eye contact. - Use plain language. - Ask patient to explain back, using their own words. - Use non-shaming, open-ended questions. - Avoid asking questions that can be answered with a simple yes or no. - Emphasise the responsibility to explain clearly is on pharmacist. - If patient unable to teach-back correctly, explain again and recheck. - Use reader-friendly print materials to support learning. - Document use of and patient response to teach-back.   **11. Counselling components for new medication and medication changes to include as appropriate:**  🞎 Drug name  🞎 Dose  🞎 Frequency of administration  🞎 Duration  🞎 Indication  🞎 Side effects  🞎 Special instructions if applicable  🞎 Reason for medication changes |
| --- |
| **12. Confirm patient’s full understanding of medication counselling using the teach-back method.** 🞎 |
| **13.**  If needed, clarify and tailor counselling, check patient recall and understanding again; repeat this process until full comprehension is achieved. |
| **14. Ask and answer patient question(s).** Ask ‘What questions do you have?’ 🞎  Do NOT ask patient ‘have you any questions?’ |
| **15. Confirm and document patient’s telephone number and a convenient time for follow-up call on data collection form.** 🞎 |
| **16. Provide patient with ‘changes to my medication list’ to take home.** 🞎 |

**Appendix 5 Changes to my Medication List**

**The medication I am allergic to:**

**Date of this form:** _dd/mm/yyyy_____________

**Completed by:** _________________________

Name MRN

DOB dd/mm/yyyy

M/F

Place Patient ID Sticker here

(This list is NOT a complete list of your medications)

| Name of medication and strength | How much medication I take? | | | | I take it every day  (Yes/No) | Why I take it? | Common/Important Side effects | Special Instructions |
| --- | --- | --- | --- | --- | --- | --- | --- | --- |
|  | **Morning/ Breakfast** | **Afternoon/Lunch** | **Evening/**  **Dinner** | **Night/**  **Bedtime** |  |  |  |  |
|  |  |  |  |  |  |  |  |  |
|  |  |  |  |  |  |  |  |  |
|  |  |  |  |  |  |  |  |  |
|  |  |  |  |  |  |  |  |  |
|  |  |  |  |  |  |  |  |  |
|  |  |  |  |  |  |  |  |  |
|  |  |  |  |  |  |  |  |  |
|  |  |  |  |  |  |  |  |  |

| **Medication to stop taking** | **Medication to take instead** |
| --- | --- |
|  |  |
|  |  |

**Appendix 6**

**Patient Telephone Survey**

Patient Initials/Study ID _______ Date of phone call ________________

Hi, my name is Emer O’Mahony, and I am a pharmacist that works at Tallaght Hospital. Is now a good time for you to talk? If *‘Yes’*: This phone call should only take a few minutes. If ‘*No*’: When would be a suitable time for me to call you back? _________________________________.

Before you were discharged from hospital, a pharmacist ________________________ had a chat with you about your medication____________________________________________________________________.

I would like to get some feedback about your experience by asking you some questions. You can ask me at any time to repeat what I have said if you cannot hear or understand me.

I will call out sentences and I will ask if you agree or disagree with each sentence. Please tell me how much you agree, disagree or if you are undecided with the following sentences.

| **Statement** | **Strongly Agree** | **Agree** | **Undecided** | **Disagree** | **Strongly Disagree** |
| --- | --- | --- | --- | --- | --- |
| 1. It is important to get information about changes to my medications before I leave hospital. |  |  |  |  |  |
| 1. It is important to get information about possible side effects of my medications before I leave hospital. |  |  |  |  |  |
| 1. Before I went home, the pharmacist__________________ spent enough time with me explaining my medications. |  |  |  |  |  |
| 1. The pharmacist explained the purpose of my medications, in a way which I could understand. |  |  |  |  |  |
| 1. The pharmacist explained how and when to take my medications at home, in a way which I could understand. |  |  |  |  |  |
| 1. Possible side effects of my medications to watch out for when I went home were explained, in a way which I could understand. |  |  |  |  |  |
| 1. Changes to my medications were explained to me in a way which I could understand**.** |  |  |  |  |  |
| 1. The written or printed information I received in hospital e.g., ‘changes to my medication list’ helped me to understand my medications when I went home. |  |  |  |  |  |
| 1. I am more confident about my knowledge of my medication after my discussion with the pharmacist in hospital. |  |  |  |  |  |

I will ask you to rate your overall experience of the information provided about your medications before you went home. Please tell me if you are satisfied, dissatisfied or undecided in response to the following question.

| **Question** | **Very Satisfied** | **Satisfied** | **Undecided** | **Dissatisfied** | **Very Dissatisfied** |
| --- | --- | --- | --- | --- | --- |
| 1. How satisfied were you with the information provided about your medication at discharge? |  |  |  |  |  |

One final question:

1. Have you any other comments to add (good or bad) about the information you received from the pharmacist about your medication before you were discharged from Tallaght Hospital?

That’s all the questions I have for you today. Thank you for your time.

**Appendix** **7**

**Pharmacist Survey**

Thank you for participating in the data collection for my MSc. I would like to obtain feedback about your experience using the teach-back method as a communication tool for discharge medication counselling.  Your response is anonymous. Please tell me how much you agree or disagree with the following statements by selecting one option only:

**1. I was given adequate information and training on how to use the teach-back method for discharge medication counselling.**

**☐ Strongly Agree**

**☐ Agree**

**☐ Neither Agree nor Disagree**

**☐ Disagree**

**☐ Strongly Disagree**

**2. The teach back method is feasible to apply in clinical practice.**

**☐ Strongly Agree**

**☐ Agree**

**☐ Neither Agree nor Disagree**

**☐ Disagree**

**☐ Strongly Disagree**

**3.Teach back is an important and effective communication method to help**
 **pharmacists ensure patients understand their medications.**

**☐ Strongly Agree**

**☐ Agree**

**☐ Neither Agree nor Disagree**

**☐ Disagree**

**☐ Strongly Disagree**

**4**. **I am confident in my ability to use teach back and I plan to use this method to provide medication counselling to my patients in the future.**

**☐ Strongly Agree**

**☐ Agree**

**☐ Neither Agree nor Disagree**

**☐ Disagree**

**☐ Strongly Disagree**

**5**. **Overall, please give feedback (positive/negative) on the discharge medication counselling process including any limitations identified. Suggestions to improve the service are welcome.**

**Appendix 8**

**Primary Discharge Diagnoses for patient participants**

| **Primary Discharge Diagnosis (ICD-10 code*)** | **Number of patients (%)** |
| --- | --- |
| Pulmonary embolism (I26) | 8 (25) |
| Atrial fibrillation (I48) | 5 (15.6) |
| Cerebral infarction (I63) | 5 (15.6) |
| Heart Failure (I50) | 2 (6.3) |
| Hypertension (I10) | 1 (3.1) |
| Type 2 diabetes mellitus (E11) | 1 (3.1) |
| Bacterial pneumonia (J15.9) | 1 (3.1) |
| Chronic obstructive pulmonary disease exacerbation (J44.1) | 1 (3.1) |
| Latent tuberculosis (Z22.7) | 1 (3.1) |
| Fibromyalgia (M79.7) | 1 (3.1) |
| Peptic ulcer (K27) | 1 (3.1) |
| Giant cell arteritis (M31.6) | 1 (3.1) |
| Transplanted organ and tissue status (Z94) | 1 (3.1) |
| Cushing syndrome (E24) | 1 (3.1) |
| Secondary thrombocytopenia (D69.5) | 1 (3.1) |
| Juvenile Arthritis (M08) | 1 (3.1) |
| Seizure (R56.8) | 1 (3.1) |
| Asthma (J45.9) | 1 (3.1) |
| Malignant neoplasm of ovary (C56) | 1 (3.1) |
| Systemic sclerosis (M34.9) | 1 (3.1) |
| Anaphylaxis, drug-induced (Y57.9) | 1 (3.1) |
| Migraine (G43) | 1 (3.1) |
| Neutropenic sepsis (A41.9) | 1 (3.1) |
| Wernicke encephalopathy (E51.2) | 1 (3.1) |

* 10th revision of the International Statistical Classification of Diseases and Related Health Problems

**Appendix 9**

**Comments from two patients dissatisfied with the medication counselling**

**Patient 18:**

- Female, aged 75 years, Charlson Comorbidity Index score of 5.
- Counselled on 5 medications for 20 minutes.
- “*The pharmacist needed to explain the information a bit more. She used jargon that professionals speak that I could not understand. I needed more time for pharmacist to explain my medication; I went home confused. My community pharmacist had to explain my medication again when I collected my new prescription*”.

**Patient 21:**

- Male, aged 81 years, Charlson Comorbidity Index score of 5.
- Counselled on 3 medications for 35 minutes.
- This patient had been readmitted to hospital at the time of the survey; the patient received written information about their medication while in hospital but did not remember this when responding to the survey.
- “*The pharmacist did not explain about my medication changes or side effects to watch out for when I went home. I suffered when I went home for 3 days. I went back into hospital because I felt unwell. I did not know that I had to take new tablets. I should have been told. I do not remember any written information given to me in the hospital. My daughter looks after all my medications*”.
